# Supplementary material for: The medial prefrontal cortex encodes procedural rules as sequential neuronal activity dynamics
Source: Mol Brain. 2025 Jul 1;18:56. doi: 10.1186/s13041-025-01230-w (PMC12220469; doi:10.1186/s13041-025-01230-w)
Supplement: Supplementary file 2 — Supplementary Material 2 [file 13041_2025_1230_MOESM2_ESM.docx]

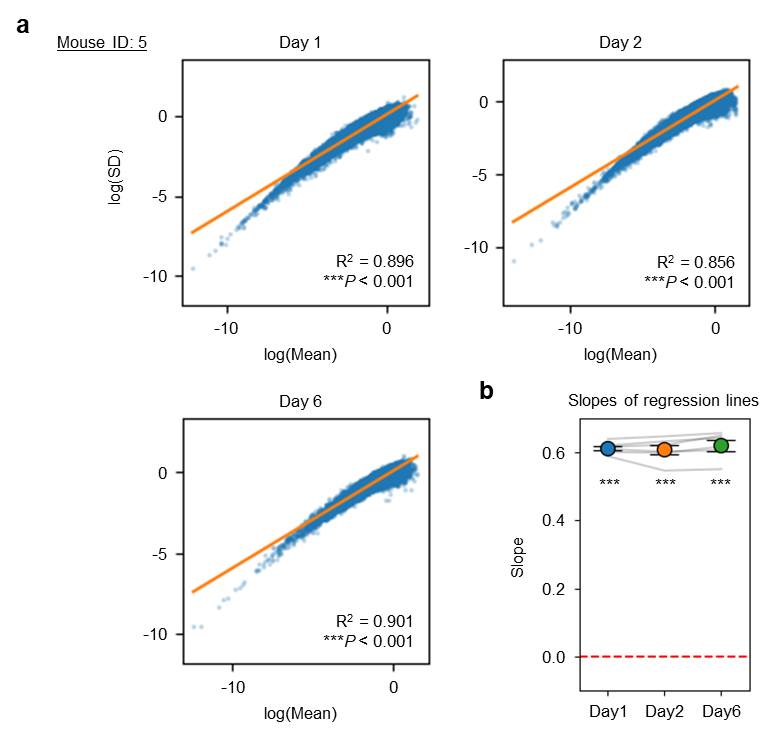


**Supplementary Fig. 1 | Ca^2+^ imaging data is influenced by signal-dependent noise. a** Examples of the correlations between the mean values and standard deviations (SD) of the data. For each minute, we calculated the mean and SD of Ca^2+^ signal intensities, then plotted these values on a logarithmic scale, as in Figure 1 of [S1]. Results for mouse ID: 5 on days 1, 2, and 6 are shown. The adjusted R^2^ and P values are shown (Wald’s test with t-distribution with Bonferroni’s correction). **b** Distribution of regression line slopes. Statistical tests were conducted to compare these slopes against zero (****P*<0.001; one-sample t-test with Bonferroni’s correction). Data are represented as mean ± SEM.


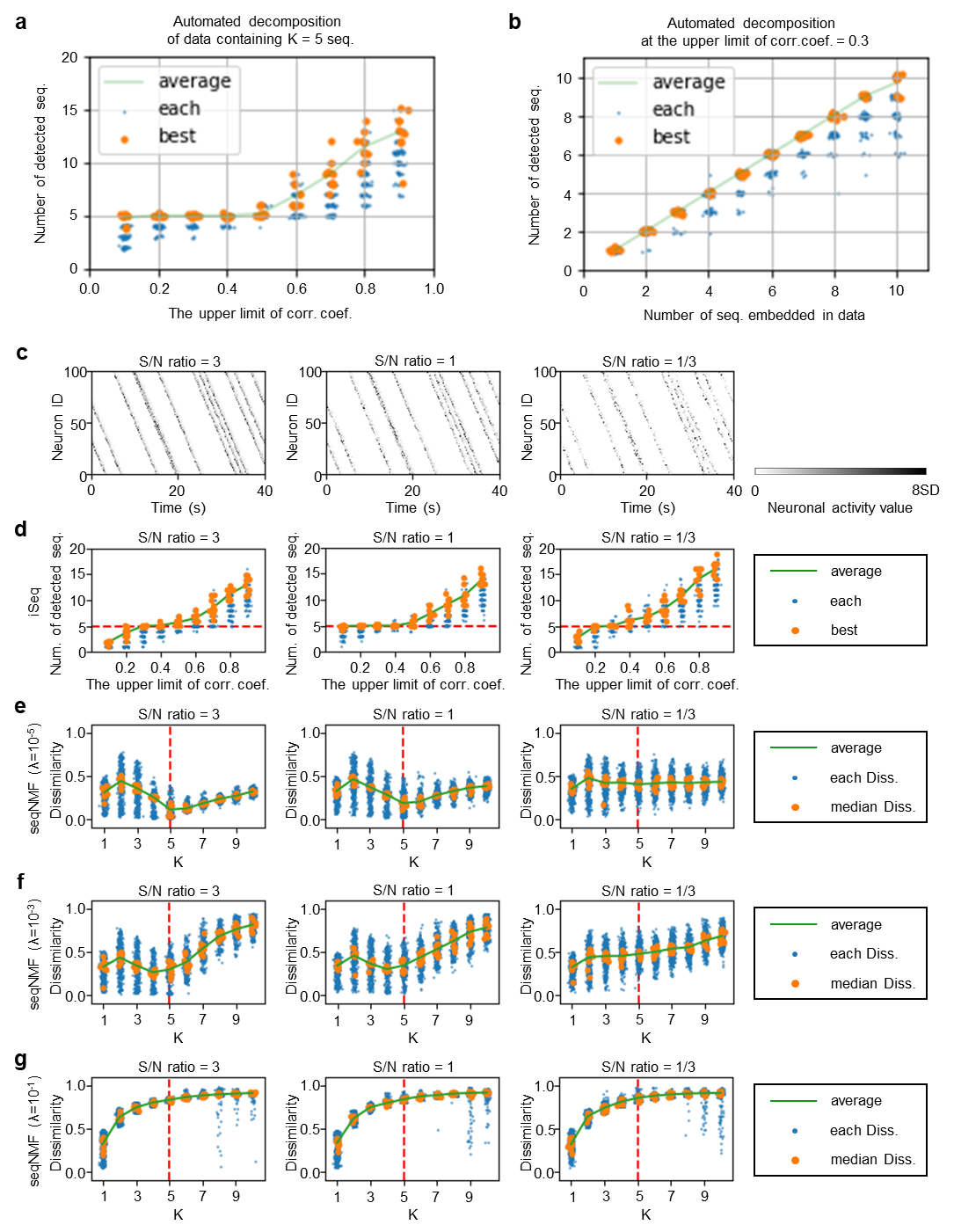


**Supplementary Fig. 2 | Validation of decomposition accuracy of iSeq and seqNMF for synthetic data. a, b** Results of decomposing synthetic data with T=30,000, conducted under the same conditions described in Fig. 1h (**a**) and 1i (**b**). **c**, Examples of neural sequences in synthetic data with different signal-to-noise ratios, generated by multiplying noise at varying levels. **d** Results of decomposing each synthetic dataset containing K=5 neuronal sequences using iSeq, following the same method as in Fig. 1h. **e–g** Synthetic data containing K=5 neuronal sequences were decomposed using seqNMF with λ values of 10^-5^ (**e**), 10^-3^(**f**), and 10^-1^(**g**), respectively. For each λ, the number of neural sequences K was varied from 1 to 9, and the dissimilarity was computed. A lower dissimilarity indicates higher consistency in the decomposition and suggests that the corresponding K value is appropriate. Abbreviations: Corr. Coef., Correlation coefficient; Seq., Neuronal sequence; S/N ratio, Signal-to-noise ratio; Num., Number; Diss., Dissimilarity.

**
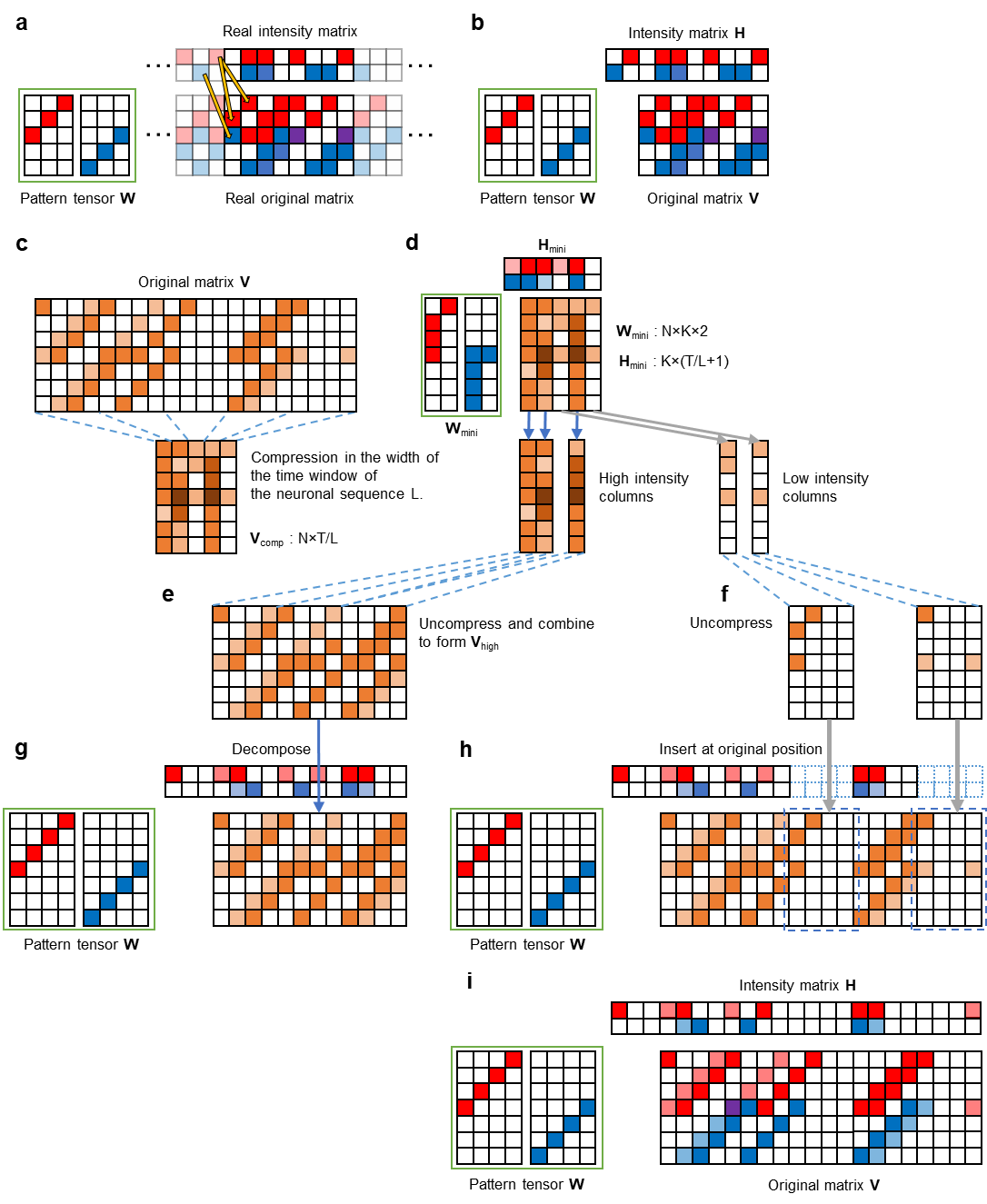
**

**Supplementary Fig. 3 | Enhancements in the accuracy and acceleration of computations in the iSeq algorithm. a** The initial portion of the original matrix **V** is influenced by neuronal sequence activities occurring outside the measured time frame. **b** The shapes of the original matrix **V**, the pattern tensor **W**, and the intensity matrix **H** in the iSeq algorithm. **c** Accelerating computations through matrix compression: the original matrix **V** is first compressed by binning according to the time window of neuronal sequence (**V**_comp_). **d V**_comp_ is decomposed into matrices **W**_mini_ and **H**_mini_. The columns of **V**_comp_ are sorted into high-intensity columns (here, top 60%) and low-intensity columns (here, bottom 40%) according to the values of **H**_mini_. **e** The high-intensity columns are decompressed and concatenated to form matrix **V**_high_. **f** The low-intensity columns are also decompressed. **g V**_high_ is decomposed to determine the pattern tensor **W**. **h** The original matrix **V** is reconstructed by inserting the decompressed matrix from (**f**) into **V**_high_. **i** With **W** held constant, the intensity matrix H is recalculated to finalize the process.

**
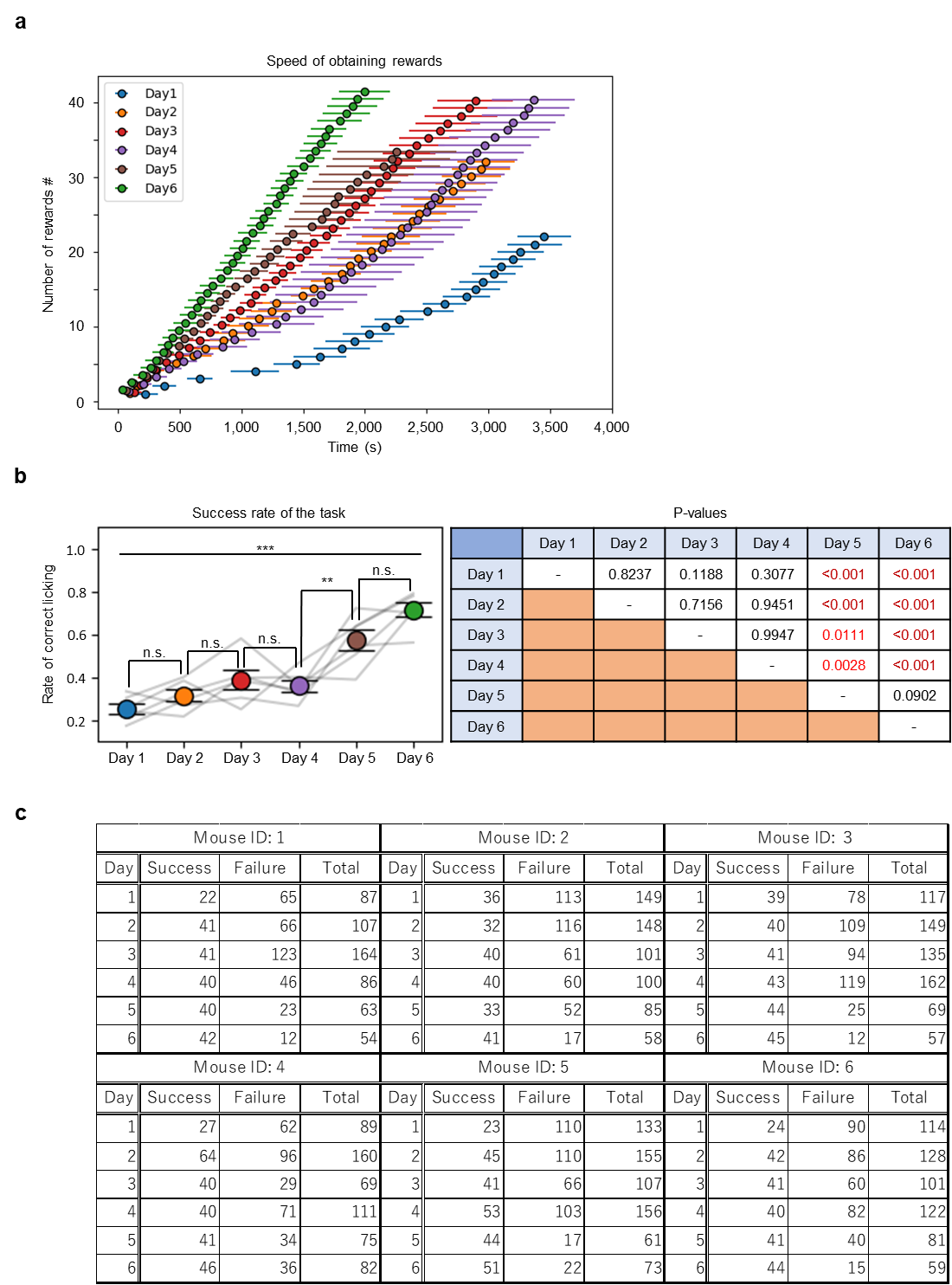
**

**Supplementary Fig. 4 | Task performance of mice during the entire training period. a** Same as Fig. 2c, but for days 1–6. **b** Same as Fig. 2d, but for days 1–6 (left). Post hoc P values (right) for all combinations of pairs (one-way RM ANOVA with Tukey’s post hoc test). **c** Numbers of Success and Failure events (licks) in each mouse and each day. Data are represented as mean ± SEM.

**
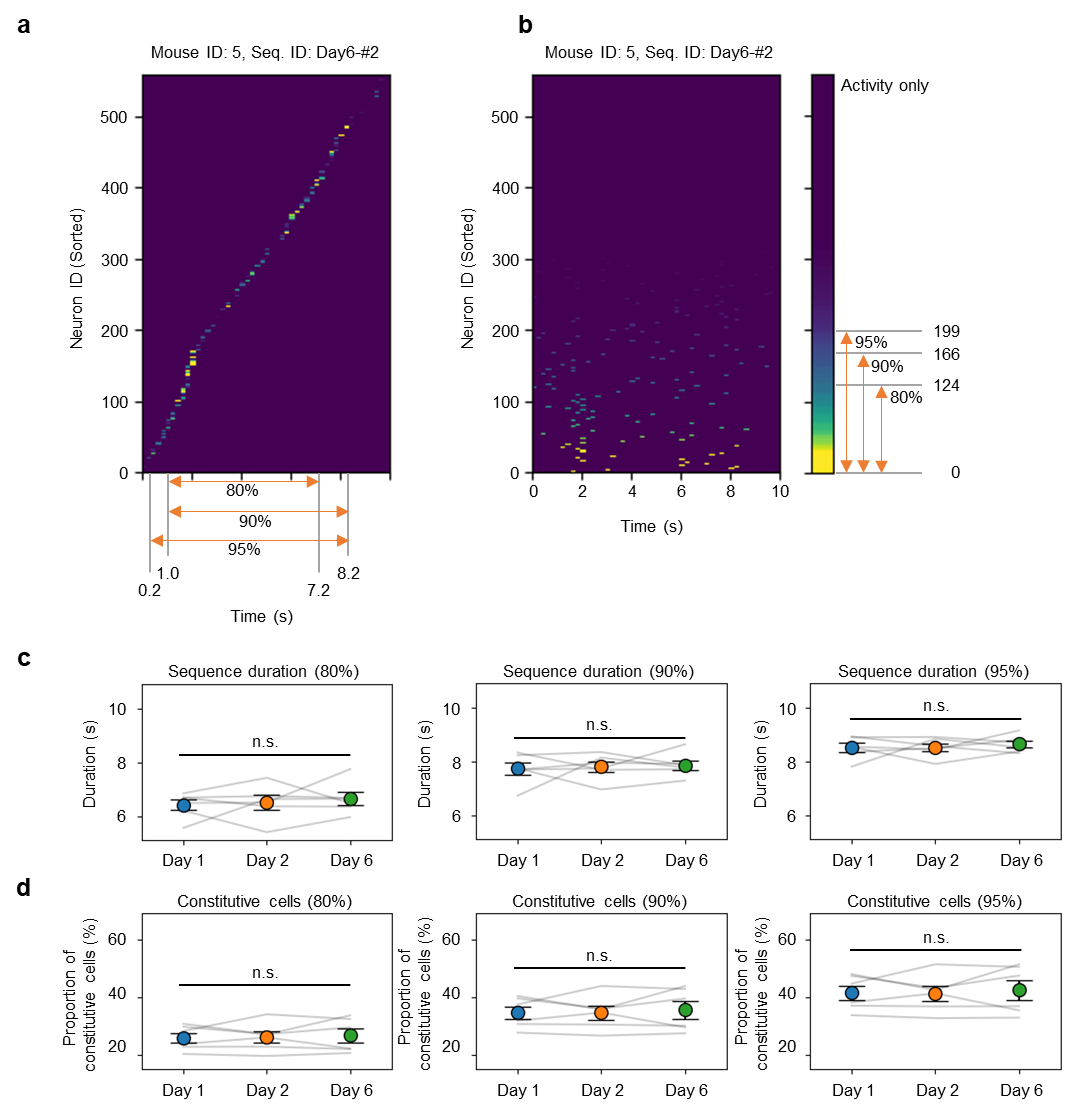
**

**Supplementary Fig. 5 | Definition of the duration and number of constitutive cells of neuronal sequences. a** Definition of duration. Neurons were sorted by the order of their activity timing within the neuronal sequence. Then the duration of the neuronal sequence was defined as the shortest period during which the sum of activity values reached 80%, 90%, and 95% of the total activity. **b** Definition of the number of constitutive cells. Neurons were sorted by the order of their activity value within the neuronal sequence. Then the number of constitutive cells was defined as the smallest number of cells for which the sum of activity values reached 80%, 90%, and 95% of the total activity. **c** Duration of detected neuronal sequences for each day of the experiment (80%, *P*=0.73; 90%, *P*=0.90; 95%, *P*=0.79; one-way RM ANOVA). **d** Same as (**c**), but for the proportion of constitutive cells relative to all cells in the measured field of Ca^2+^ imaging (80%, *P*=0.81; 90%, *P*=0.79; 95%, *P*=0.80; one-way RM ANOVA). These calculations were performed after the sequence-sharpening operation (see Supplementary Fig. 6**d, e**). Abbreviations: Seq., Neuronal sequence. Data are represented as mean ± SEM.

**
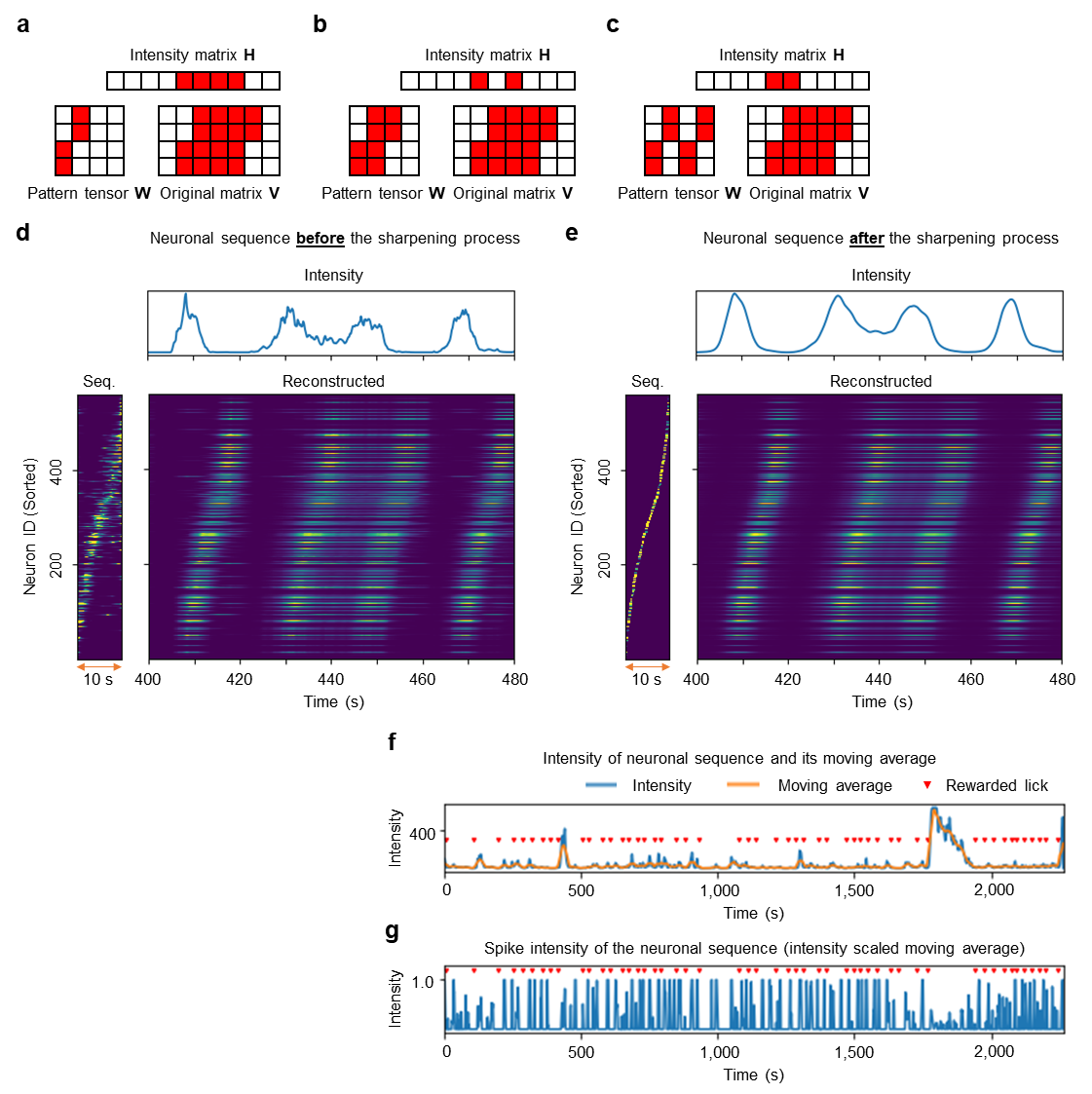
**

**Supplementary Fig. 6 | The sequence-sharpening operation and spike intensity. a–c** An example of the same original matrix **V** being decomposed into (**a**) “narrow”, (**b**) “wider”, and (**c**) “separated” sequences. **d, e** An example of before (**d**) and after (**e**) the sequence-sharpening operation. The following is shown: the intensity of the neuronal sequence (top), the shape of the neuronal sequence (bottom left), and the neuronal activity reconstructed from the shape and intensity of the neuronal sequence (bottom right). **f** An example of the intensity of a neuronal sequence and its moving average. **g** The spike intensity calculated from (**f**). Abbreviations: Seq., Neuronal sequence.

**
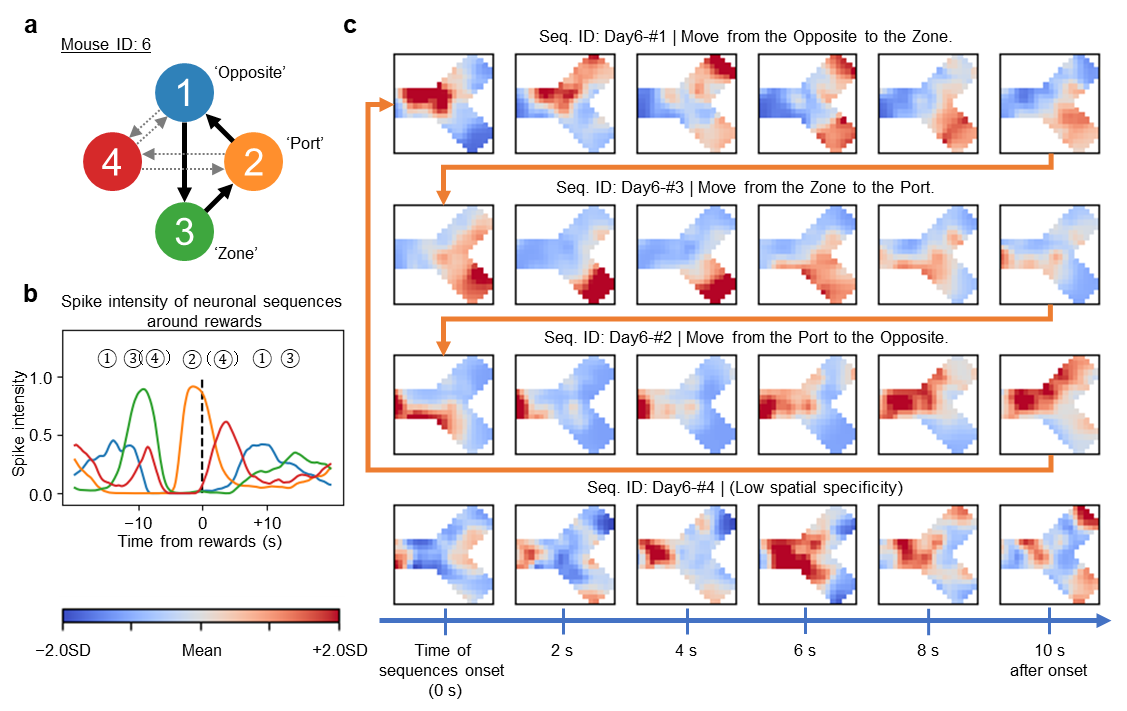
**

**Supplementary Fig. 7 | Neuronal sequences of the mouse with a three-step action comprehension of the rule. a** The relations between neuronal sequences and rule components of mouse ID: 6. **b** Time series of mean spike intensities of neuronal sequences before and after obtaining a reward. The number at the top represents the corresponding sequence ID. **c** Same as Fig. 4**d**, but for mouse ID: 6. Abbreviations: Seq., Neuronal sequence.


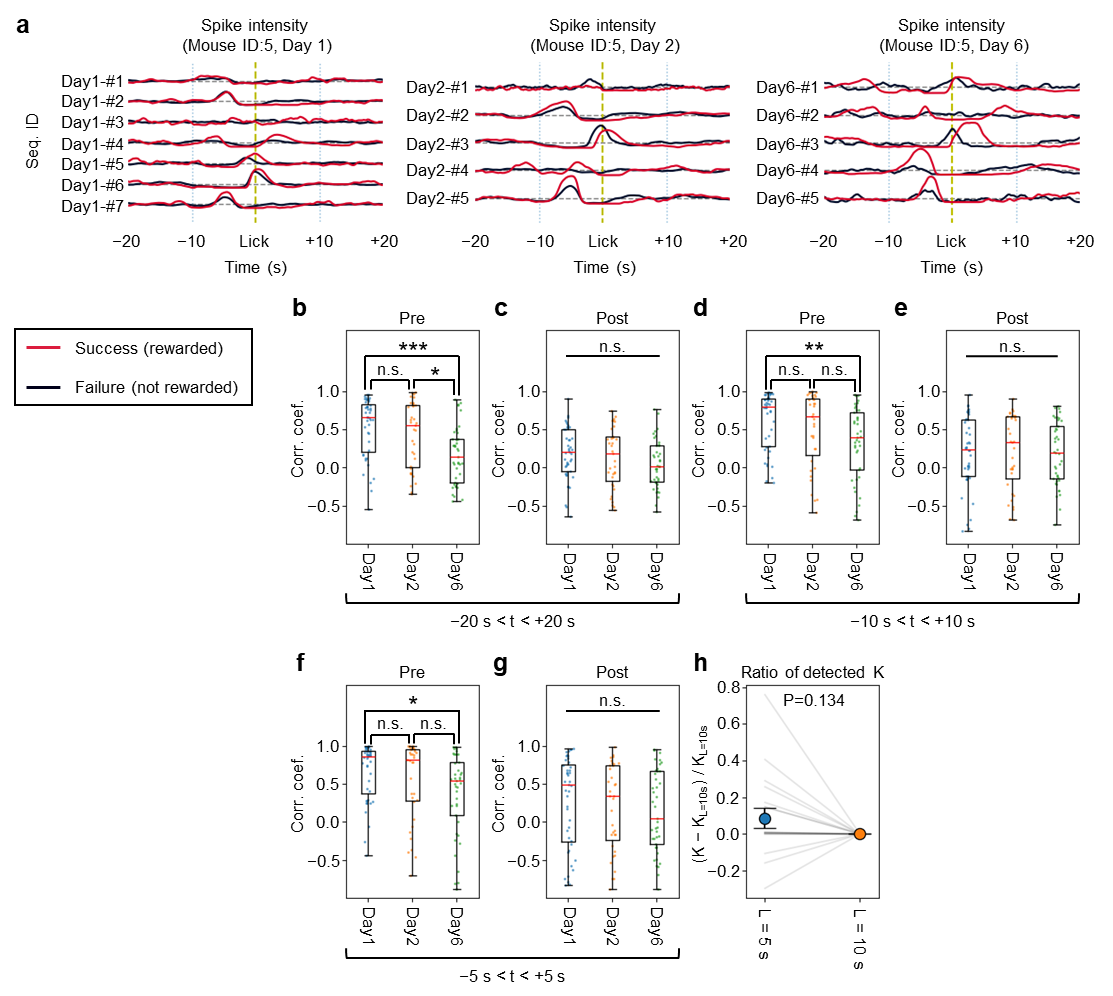


**Supplementary Fig. 8 | The neuronal sequences of expert mice detected with a time window of L = 5 s also distinguish between successful and unsuccessful reward acquisition.** Except for the use of a 5-second time window L in iSeq, **a** corresponds to Fig. 5b, **b** corresponds to Fig. 5d (****P*<0.001; **P*=0.0135; one-way ANOVA with Tukey’s post hoc test), **c** corresponds to Fig. 5e, d corresponds to Fig. 5f (***P*=0.0076), and e corresponds to Fig. 5g (***P*<0.001), **d** corresponds to Fig. 5f. **f** Same as (**b**), but focused on the 5 s prior to licking (**P*=0.0138). **g** Same as (**c**), but focused on the 5 s after licking. **h** The relative increase or decrease in the number of neuronal sequences K for each mouse and each day, normalised to K detected with L = 10 s (one-sample t-test).


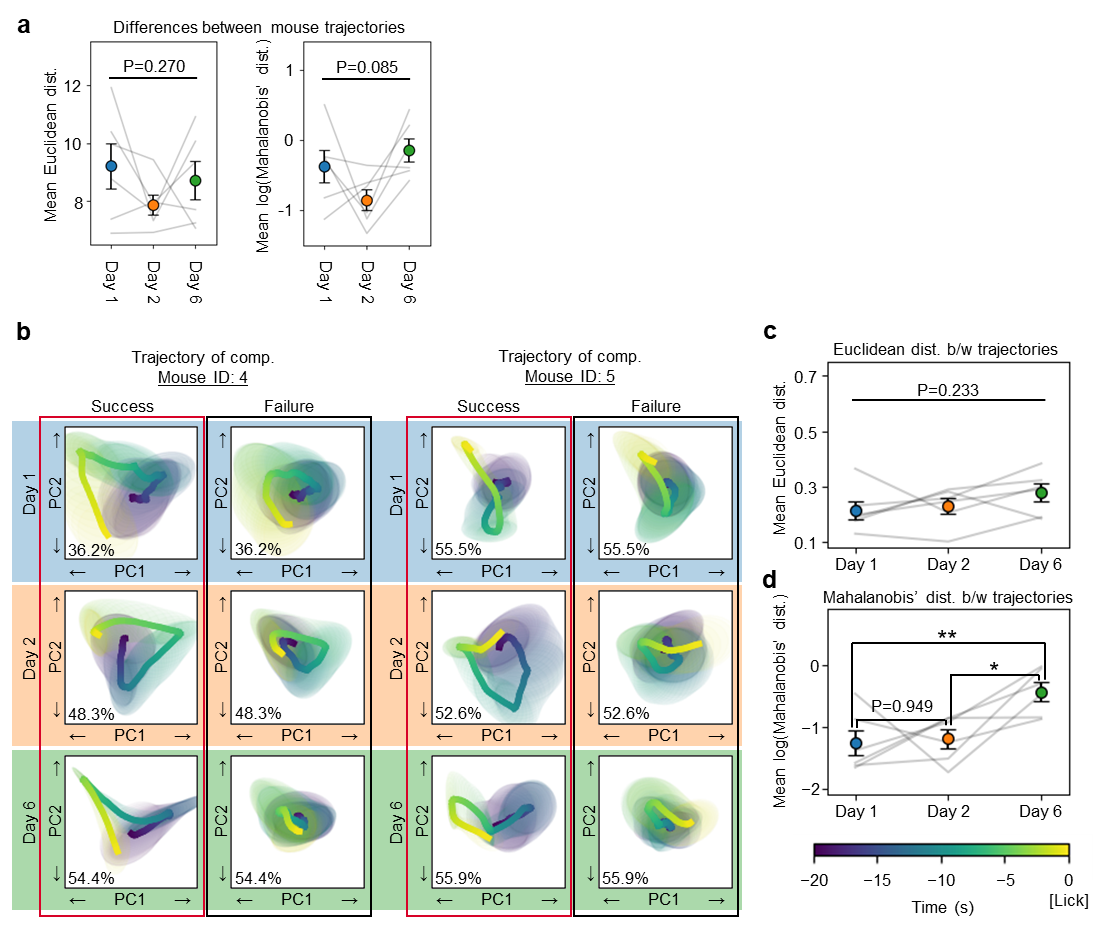


**Supplementary Fig. 9 | Even when trajectory distances were computed using pooled data from both Success and Failure, the same conclusion as in Fig. 6 held.** **a** Same as Fig. 2g-left (left) and same as Fig. 2g-right, but Mahalanobis’ distances were calculated based on the pooled covariance (right). **b** Same as Fig. 6d, but Procrustes alignments were carried out based on the average trajectory across both Success and Failure. **c** Same as Fig. 6e. **d** Same as Fig. 6f, but Mahalanobis’ distances were calculated based on the pooled covariance. Abbreviations: Seq., Neuronal sequence; PC, Principal component; Dist., Distance; b/w, Between. Data are represented as mean ± SEM.


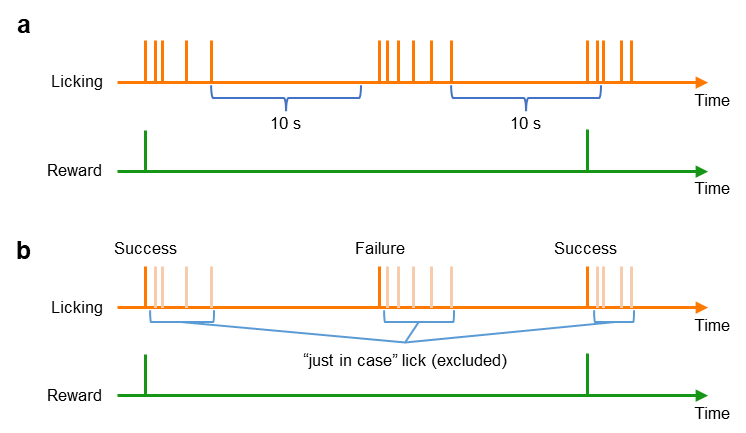


**Supplementary Fig. 10 | Schema illustrating the classification of Success and Failure events. a** Example timeline of licking events and reward acquisition. **b** Result of applying the event classification algorithm: Success and Failure events are identified, and licking events classified as “just-in-case” are excluded.


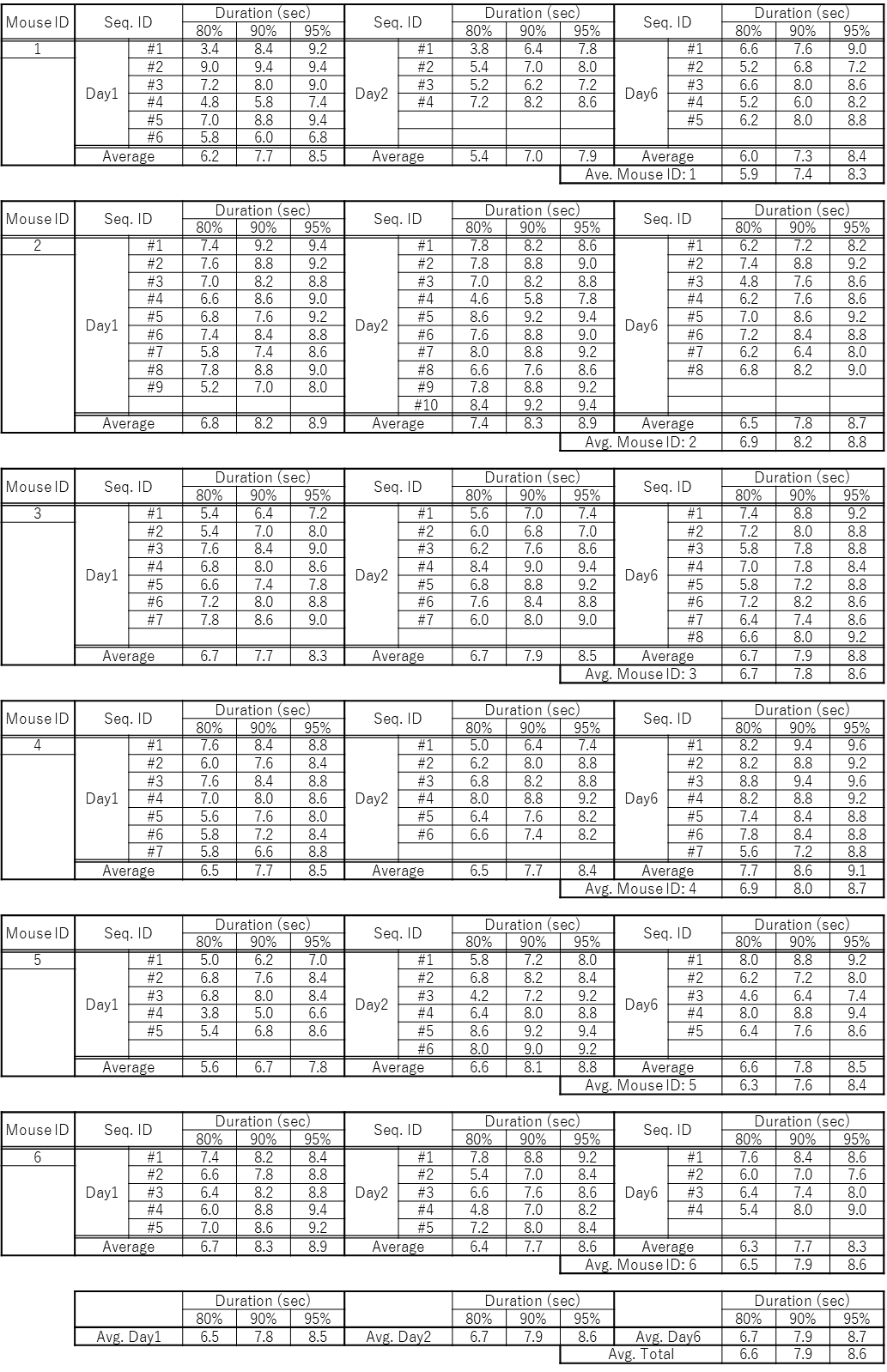


**Supplementary Table 1 | The duration of neuronal sequences.**

**
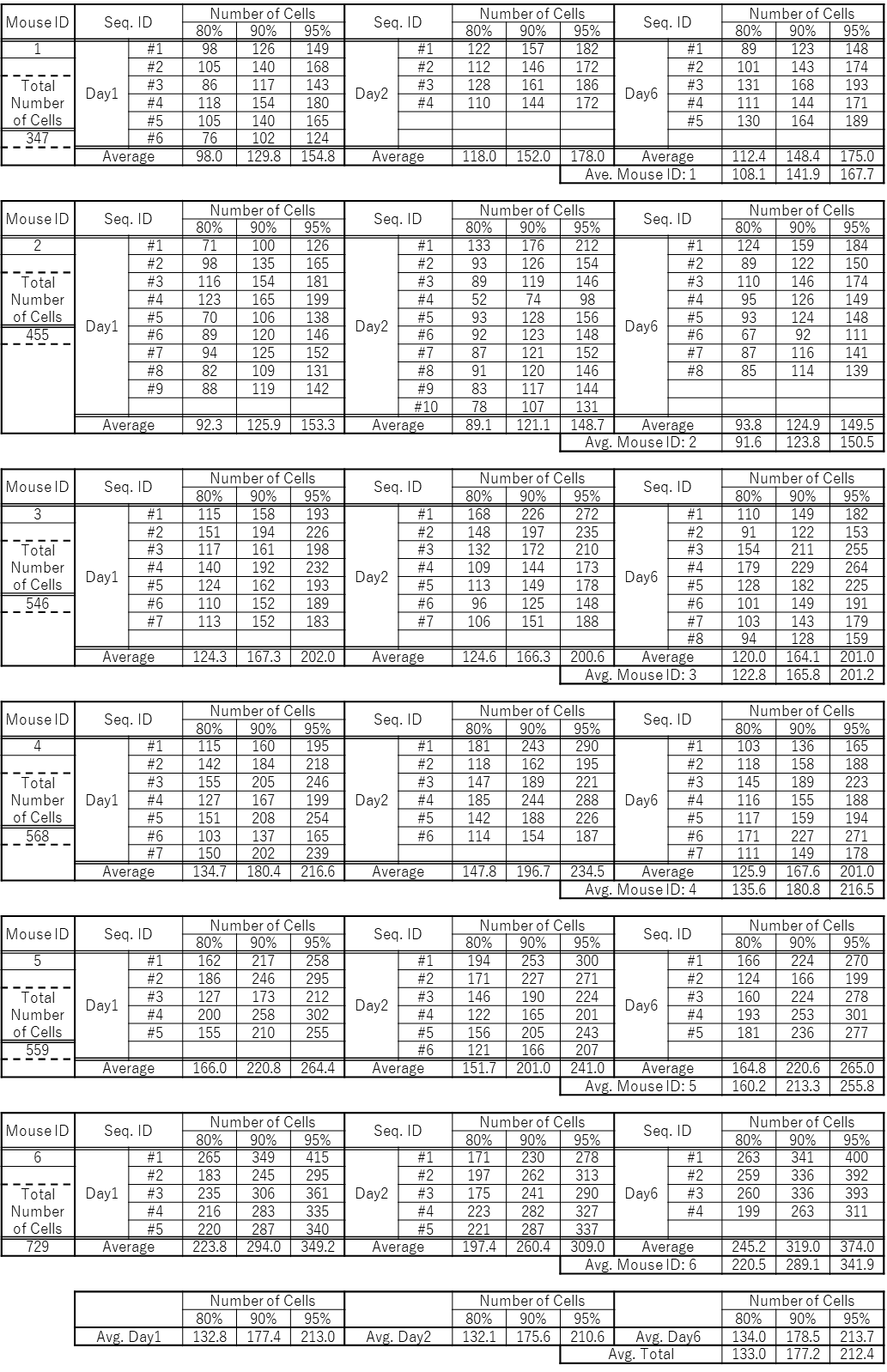
**

**Supplementary Table 2 | The number of constitutive cells of neuronal sequences.**

**Supplementary Math Note 1 | Definitions of operators used in this paper.**

According to Mackevicius et al. 2019 [S2], we defined each operator as follows.

**Matrix indexing.** Any index of a vector with length T is specified by $t$. This case relationship applies to all letters. Additionally, when indexing specifically extracts elements of a matrix, the elements are represented by the lower-case letter corresponding to the letter representing the matrix. In other words, the element in row $n$ and column $t$ of the original matrix **V** is $v_{nt}$.

To accommodate negative indexing, which is discussed below, 0-based indexing is applied throughout this article. This means that vectors and rows and columns of matrices of length T are assigned an index number from 0 to T−1. In addition, we use $\cdot$ to represent all elements along each dimension of the matrix. Therefore, $\mathbf{V}_{\cdot t}$ means the vector of $\left\{ v_{0,t}, v_{1,t}, \cdots, v_{N-1,t} \right\}$. For the pattern tensor **W**, which is a 3D tensor, $\mathbf{W}_{\cdot k\cdot}$ is a 2D matrix and represents the shape of the k^th^ neuronal sequence.

The intensity matrix **H** is partially negatively indexed because it is extended forward in the time dimension by L−1. This means that the range of indices in the time dimension of **H** is −L+1 to T−1.

**Shift operator.** The operator $\overset{\boldsymbol{l\to}}{\mathbf{V}}$ shifts $\mathbf{V}$ in the direction of $\to$ by $l$ time frames. In other words, the correspondence is as follows:

$$\left( \overset{l\to}{\mathbf{V}} \right)_{\cdot t}=\mathbf{V}_{\cdot\left( t-l \right)}$$

$$\left( \overset{\leftarrow l}{\mathbf{V}} \right)_{\cdot t}=\mathbf{V}_{\cdot\left( t+l \right)}$$

The shift operator returns 0 if the reference is outside the range of the matrix’s index.

**Tensor convolution operator.** This computes a 2D matrix from a 3D tensor and a 2D matrix. From the pattern tensor **W** and intensity matrix **H**, reconstructed matrix **U** is obtained. The formula is as follows:

$$\mathbf{U}=\mathbf{W⊛H}=\sum_{l=0}^{L-1} \mathbf{W}_{\cdot\cdot l}\overset{l\to}{\mathbf{H}}$$

Each element of **U** can be calculated as follows, each reconstructed by the sum of $k$ convolutions:

$$u_{nt}=\sum_{k=0}^{K-1} \sum_{l=0}^{L-1} w_{nkl}h_{k(t-l)}\equiv\left( \mathbf{W}⊛\mathbf{H} \right)_{nt}$$

**Transpose tensor convolution operator.** This computes a 2D matrix from a 3D tensor and a 2D matrix. From the pattern tensor **W** and original matrix **V**, the overlap matrix **R** is obtained. The formula is as follows:

$$\mathbf{R}=\mathbf{W}\overset{\top}{⊛}\mathbf{V}=\sum_{l=0}^{L-1} \left( \mathbf{W}_{\cdot\cdot l} \right)^{\top}\overset{\leftarrow l}{\mathbf{V}}$$

Each element of **R** can be calculated as follows: each measures the overlap (correlation) between the data and $k$^th^ neuronal sequence at time $t$.

$$r_{kt}=\sum_{n=0}^{N-1} \sum_{l=0}^{L-1} w_{nkl}x_{n(t+l)}\equiv\left( \mathbf{W}\overset{\top}{⊛}\mathbf{V} \right)_{kt}$$

**Supplementary Math Note 2 | Corrections to guarantee convergence of the multiplicative update rules.**

**Derivation of the multiplicative update rules.** The error in approximating the original matrix **V** with the reconstructed matrix **U** is calculated based on Itakura-Saito divergence as follows:

$$D\left( \mathbf{V}, \mathbf{U} \right)=\sum_{n=0}^{N-1} \sum_{t=0}^{T-1} \frac{v_{nt}}{u_{nt}}-\log\frac{v_{nt}}{u_{nt}}-1$$

Because each element of **U** is calculated from the pattern tensor **W** and intensity matrix **H** as follows (see Supplementary Equation 1):

$$u_{nt}=\sum_{k=0}^{K-1} \sum_{l=0}^{L-1} w_{nkl}h_{k(t-l)}.$$

When $u_{nt}$ is partially differentiated with respect to $w_{nkl}$ and $h_{k(t-l)}$, the following expressions are obtained:

$$\frac{\partial u_{nt}}{\partial w_{nkl}}=h_{k\left( t-l \right)}$$

$$\frac{\partial u_{nt}}{\partial h_{k\left( t-l \right)}}=w_{nkl}.$$

Similarly, the partial derivative of $D\left( \mathbf{V},\mathbf{U} \right)$ with respect to $u_{nt}$ is given as follows:

$$\frac{\partial D\left( \mathbf{V, U} \right)}{\partial u_{nt}}=\frac{1}{u_{nt}}-\frac{v_{nt}}{u_{nt}^{2}}.$$

Therefore, by the chain rule, differentiating $D\left( \mathbf{V},\mathbf{U} \right)$ by $w_{nkl}$ or $h_{kt}$ yields the following gradients:

$$\frac{\partial D\left( \mathbf{V},\mathbf{U} \right)}{\partial w_{nkl}}=\sum_{t=0}^{T-1} \frac{\partial D\left( \mathbf{V},\mathbf{U} \right)}{\partial u_{nt}}\cdot\frac{\partial u_{nt}}{\partial w_{nkl}}=\sum_{t=0}^{T-1} \left( \frac{1}{u_{nt}}-\frac{v_{nt}}{u_{nt}^{2}} \right)h_{k\left( t-l \right)}$$

$$\frac{\partial D\left( \mathbf{V},\mathbf{U} \right)}{\partial h_{kt}}=\sum_{n=0}^{N-1} \sum_{l=0}^{L-1} \frac{\partial D\left( \mathbf{V},\mathbf{U} \right)}{\partial u_{n(t+l)}}\cdot\frac{\partial u_{n(t+l)}}{\partial h_{kt}}=\sum_{n=0}^{N-1} \sum_{l=0}^{L-1} w_{nkl}\left( \frac{1}{u_{n(t+l)}}-\frac{v_{n(t+l)}}{u_{n(t+l)}^{2}} \right)$$

Furthermore, this can be expressed in matrix form as:

$$\frac{dD\left( \mathbf{V},\mathbf{U} \right)}{d\mathbf{W}_{\cdot\cdot l}}=\frac{\mathbf{1}}{\mathbf{U}}\left( \overset{l\to}{\mathbf{H}} \right)^{\top}-\frac{\mathbf{V}}{\mathbf{U}^{2}}\left( \overset{l\to}{\mathbf{H}} \right)^{\top}$$

$$\frac{dD\left( \mathbf{V},\mathbf{U} \right)}{d\mathbf{H}}=\mathbf{W}\overset{\top}{⊛}\frac{\mathbf{1}}{\mathbf{U}}-\mathbf{W}\overset{\top}{⊛}\frac{\mathbf{V}}{\mathbf{U}^{2}}$$

From these gradients and the appropriate learning rates $\eta_{\mathbf{W}}$ and $\eta_{\mathbf{H}}$, the additive update rules are derived.

$$\mathbf{W}_{\cdot\cdot l}^{t+1}=\mathbf{W}_{\cdot\cdot l}^{t}-\eta_{\mathbf{W}}\frac{dD\left( \mathbf{V},\mathbf{U}^{t} \right)}{d\mathbf{W}_{\cdot\cdot l}^{t}}=\mathbf{W}_{\cdot\cdot l}^{t}-\eta_{\mathbf{W}}\left\{ \frac{\mathbf{1}}{\mathbf{U}^{t}}\left( \overset{l\to}{\mathbf{H}^{t}} \right)^{\top}-\frac{\mathbf{V}}{\left( \mathbf{U}^{t} \right)^{2}}\left( \overset{l\to}{\mathbf{H}^{t}} \right)^{\top} \right\}$$

$$\mathbf{H}^{t+1}=\mathbf{H}^{t}-\eta_{\mathbf{H}}\frac{dD\left( \mathbf{V},\mathbf{U}^{t} \right)}{d\mathbf{H}^{t}}=\mathbf{H}^{t}-\eta_{\mathbf{H}}\left( \mathbf{W}^{t}\overset{\top}{⊛}\frac{\mathbf{1}}{\mathbf{U}^{t}}-\mathbf{W}^{t}\overset{\top}{⊛}\frac{\mathbf{V}}{\left( \mathbf{U}^{t} \right)^{2}} \right)$$

Here, by setting $\eta_{\mathbf{W}}=\frac{\mathbf{W}_{\cdot\cdot l}^{t}}{\frac{\mathbf{1}}{\mathbf{U}^{t}}\left( \overset{l\to}{\mathbf{H}^{t}} \right)^{\top}}$ and $\eta_{\mathbf{H}}=\frac{\mathbf{H}^{t}}{\mathbf{W}^{t}\overset{\top}{⊛}\frac{\mathbf{1}}{\mathbf{U}^{t}}}$, the multiplicative rules are derived.

$$\mathbf{W}_{\cdot\cdot l}^{t+1}=\mathbf{W}_{\cdot\cdot l}^{t}\times\frac{\frac{\mathbf{V}}{\left( \mathbf{U}^{t} \right)^{2}}\left( \overset{l\to}{\mathbf{H}^{t}} \right)^{\top}}{\frac{\mathbf{1}}{\mathbf{U}^{t}}\left( \overset{l\to}{\mathbf{H}^{t}} \right)^{\top}}$$

$$\mathbf{H}^{t+1}=\mathbf{H}^{t}\times\frac{\mathbf{W}^{t}\overset{\top}{⊛}\frac{\mathbf{V}}{\left( \mathbf{U}^{t} \right)^{2}}}{\mathbf{W}^{t}\overset{\top}{⊛}\frac{\mathbf{1}}{\mathbf{U}^{t}}}$$

The multiplicative update rules ensure that the elements of the tensor **W** and the matrix **H** are non-negative. However, the learning rates are fixed in this process, and a case that is too large can be expected, resulting in an increase in the reconstruction error $D\left( \mathbf{V},\mathbf{U} \right)$ (i.e., $D\left( \mathbf{V}, \mathbf{U}^{t+1} \right)>D\left( \mathbf{V},\mathbf{U}^{t} \right)$).

**Reducing the learning rates.** Consider multiplying learning rates by $\alpha\left( 0<\alpha<1 \right)$, respectively. The additive update rule for the intensity matrix **H** is then as follows:

$$\mathbf{H}^{t+1}=\mathbf{H}^{t}-\alpha\eta_{\mathbf{H}}\left( \mathbf{W}^{t}\overset{\top}{⊛}\frac{\mathbf{1}}{\mathbf{U}^{t}}-\mathbf{W}^{t}\overset{\top}{⊛}\frac{\mathbf{V}}{\left( \mathbf{U}^{t} \right)^{2}} \right)$$

$$=\left( 1-\alpha\right)\mathbf{H}^{t}+\alpha\times\mathbf{H}^{t}\times\frac{\mathbf{W}^{t}\overset{\top}{⊛}\frac{\mathbf{V}}{\left( \mathbf{U}^{t} \right)^{2}}}{\mathbf{W}^{t}\overset{\top}{⊛}\frac{\mathbf{1}}{\mathbf{U}^{t}}},$$

where the second term is equal to **H**^t+1^ when the learning rate is $\eta_{\mathbf{H}}$ (i.e., when $\alpha=1$), except that $\alpha$ is multiplied. Therefore, since $0<\alpha<1$, the second term is non-negative. The first term is also non-negative, and thus the whole remains non-negative. The same holds for the update rule for pattern tensor **W**. From the above, the following replacements can be applied to reduce the learning rates while guaranteeing that the matrices are non-negative.

$$\mathbf{W}^{t+1}\leftarrow\left( 1-\alpha\right)\mathbf{W}^{t}+\alpha\mathbf{W}^{t+1}$$

$$\mathbf{H}^{t+1}\leftarrow\left( 1-\alpha\right)\mathbf{H}^{t}+\alpha\mathbf{H}^{t+1}$$

This replacement can be repeated m times to increase the learning rates by a factor of $\alpha^{m}$, i.e.,

$$\left( 1-\alpha^{m} \right)\mathbf{H}^{t}+\alpha^{m}\mathbf{H}^{t+1}=\left( 1-\alpha\right)\mathbf{H}^{t}+\alpha\left\{ \left( 1-\alpha^{m-1} \right)\mathbf{H}^{t}+\alpha^{m-1}\mathbf{H}^{t+1} \right\}$$

We therefore repeated this replacement until $D\left( \mathbf{V},\mathbf{U}^{t+1} \right)$ was smaller than $D\left( \mathbf{V},\mathbf{U}^{t} \right)$.

**Supplementary Math Note 3 | Derivation of the prediction (decoding) error coefficients for “X-coordinate” and “Y-coordinate”.**

We defined the prediction (decoding) error function for “X-coordinate” and “Y-coordinate” as follows:

$$E=\alpha\sum_{t\in Z_{n}} \left( z\left( t \right)-\tilde{z}\left( t \right) \right)^{2}+\beta\sum_{t\in Z_{p}} \left( z\left( t \right)-\tilde{z}\left( t \right) \right)^{2}$$

$$Z_{n}=\left\{ t|z\left( t \right)\leq0.5 \right\}, Z_{p}=\left\{ t|z\left( t \right)>0.5 \right\}.$$

Here, we consider a decoder that always outputs a constant value $\tilde{z}\left( t \right)=c$, and denote the resulting error as $E\left( c \right)$:

$$E\left( c \right)=\alpha\sum_{t\in Z_{n}} \left( z\left( t \right)-c \right)^{2}+\beta\sum_{t\in Z_{p}} \left( z\left( t \right)-c \right)^{2}.$$

Let

$$Q_{n}=\sum_{t\in Z_{n}} z\left( t \right), R_{n}=\sum_{t\in Z_{n}} {z\left( t \right)}^{2}, Q_{p}=\sum_{t\in Z_{p}} z\left( t \right), R_{p}=\sum_{t\in Z_{p}} {z\left( t \right)}^{2}$$

, $E\left( c \right)$ and its first derivative can be expressed as follows:

$$E\left( c \right)=\alpha\left( R_{n}-2cQ_{n}+\left| Z_{n} \right|c^{2} \right)+\beta\left( R_{p}-2cQ_{p}+\left| Z_{p} \right|c^{2} \right)$$

$$\frac{dE}{dc}\left( c \right)=-2\alpha\left( Q_{n}+\left| Z_{n} \right|c \right)-2\beta\left( Q_{p}+\left| Z_{p} \right|c \right).$$

Under the constraint $\tilde{z}\left( t \right)=c$, we consider the decoder that yields the minimal possible error—referred to here as the null decoder. If this decoder is defined such that it attains the minimum value $0.25\left| Z \right|$ when $c=0.5$, then the condition is written as follows:

$$\left\{ \begin{aligned} E\left( 0.5 \right)=0.25\left| Z \right| \\ \frac{dE}{dc}\left( 0.5 \right)=0 \end{aligned} \right..$$

This leads to the following system of equations:

$$\left\{ \begin{aligned} \alpha\left( 4R_{n}-4Q_{n}+\left| Z_{n} \right| \right)+\beta\left( 4R_{p}-4Q_{p}+\left| Z_{p} \right| \right)=\left| Z \right| \\ \alpha\left( 2Q_{n}+\left| Z_{n} \right| \right)+\beta\left( 2Q_{p}+\left| Z_{p} \right| \right)=0 \end{aligned} \right..$$

From the first equation, we isolate $\alpha$ to obtain

$$\alpha=\frac{\left| Z \right|-\beta\left( 4R_{p}-4Q_{p}+\left| Z_{p} \right| \right)}{4R_{n}-4Q_{n}+\left| Z_{n} \right|}$$

, and substituting this result into the second equation leads to the conclusion that

$$\beta=\frac{\left| Z \right|\left( \left| Z_{n} \right|-2Q_{n} \right)}{8\left( Q_{p}R_{n}-Q_{n}R_{p} \right)-4\left| Z_{n} \right|\left( Q_{p}-R_{p} \right)+4\left| Z_{p} \right|\left( Q_{n}-R_{n} \right)-2\left( Q_{n}\left| Z_{p} \right|-Q_{p}\left| Z_{n} \right| \right)}.$$

**Supplementary References**

[S1] Devarajan, K. & Cheung, V. C. K. On Nonnegative Matrix Factorization Algorithms for Signal-Dependent Noise with Application to Electromyography Data. *Neural Computation* 2014;26:6:1128-1168:<https://doi.org/10.1162/NECO_a_00576>.

[S2] Mackevicius, E. L. *et al.* Unsupervised discovery of temporal sequences in high-dimensional datasets, with applications to neuroscience. *eLife* 2019;8:e38471:<https://doi.org/10.7554/eLife.38471>.
